# High H_2_O Content in Pyroxenes of Residual Mantle Peridotites at a Mid Atlantic Ridge Segment

**DOI:** 10.1038/s41598-019-57344-4

**Published:** 2020-01-17

**Authors:** Pei Li, Qun-Ke Xia, Luigi Dallai, Enrico Bonatti, Daniele Brunelli, Anna Cipriani, Marco Ligi

**Affiliations:** 1 School of Earth Sciences, Zhejiang University, Hangzhou, 310027 China; 2 Istituto di Geoscienze e Georisorse-CNR, Via G. Moruzzi 1, 56124 Pisa, Italy; 3 Lamont-Doherty Earth Observatory of Columbia University, Palisades, New York, 10964 USA; 4 Istituto di Scienze Marine-CNR, via Gobetti 101, 40129 Bologna, Italy; 5 Dipartimento di Scienze Chimiche e Geologiche, Università di Modena e Reggio Emilia, Modena, 41100 Italy

**Keywords:** Geology, Geochemistry

## Abstract

Global correlations of mid-ocean-ridges basalt chemistry, axial depth and crustal thickness have been ascribed to mantle temperature variations affecting degree of melting. However, mantle H_2_O content and elemental composition may also play a role. How H_2_O is distributed in the oceanic upper mantle remains poorly constrained. We tackled this problem by determining the H_2_O content of orthopyroxenes (opx) and clinopyroxenes (cpx) of peridotites from a continuous lithospheric section created during 26 Ma at a 11°N Mid-Atlantic Ridge segment, and exposed along the Vema Transform. The H_2_O content of opx ranges from 119 ppm to 383 ppm; that of cpx from 407 ppm to 1072 ppm. We found anomalous H_2_O-enriched peridotites with their H_2_O content not correlating inversely with their degree of melting, although H_2_O is assumed to be incompatible during melting. Inverse correlation of H_2_O with Ce, another highly incompatible component, suggests post-melting H_2_O enrichment. We attribute a major role to post-melting temperature-dependent diffusion of hydrogen occurring above the melting region, where water-rich melt flows faster than residual peridotites through dunitic conduits cross-cutting the uprising mantle. Accordingly, estimates of the H_2_O content of the MORB mantle source based on H_2_O in abyssal peridotites can be affected by strong uncertainties.

## Introduction

Mantle material upwelling below mid-oceanic ridges undergoes decompression melting; the melt rises and cools to form the crust, while the melting residue forms the lithospheric mantle. It has long been held that the mantle that rises under ridges is more or less uniform in chemistry but varies in temperature by hundreds of degrees^1–4^. It would follow that the correlated variability among topography, structure and composition observed along the global mid-oceanic ridge system is ultimately controlled by the temperature of the underlying mantle^1–4^. However, there is growing evidence for a non-thermal influence on mantle melting processes beneath ridges, such as at Galapagos and Azores^5,6^. But how volatiles, such as water, and other compositional heterogeneities, are distributed in the sub-ridge mantle, and how they affect the physical and chemical properties of mid-ocean ridges, remains poorly constrained.

An uplifted sliver of lithosphere exposed at 11°N along the Vema transform (Vema Lithospheric Section or VLS), representing a 26 Ma time interval of creation of lithosphere at a segment of Mid Atlantic Ridge^7,8^ (MAR), provides an opportunity to study variations through time of composition and thermal state of the sub-ridge upper mantle. Degree of melting and crustal thickness show 3–4 Ma oscillations superimposed on a long-term steady increase with time, interpreted as due to mantle thermal variations^8–10^. However, radiogenic isotopes suggest that the extent of melting was also affected by mantle chemical heterogeneities^11,12^.

In this study, we estimated the H_2_O content of nominally anhydrous minerals of the sub-ridge melting source in order to assess its potential effect on mantle melting variations. We determined the H_2_O content of eighteen mantle-derived abyssal peridotites recovered from ~10 closely spaced sites along the basal unit of the VLS. The crustal age of these sites, estimated from magnetic anomalies and basaltic glass ^40^Ar/^39^Ar dating, ranges from 19.2 to 10.2 Ma^8,11,13^. The VLS peridotites chosen for this study have been the object of previous studies^8–12^; they display protogranular/porphyroclastic textures and their relict mineral phases include orthopyroxene (opx), clinopyroxene (cpx), spinel (sp), and rare olivine^8,9,11^; they are serpentinized to various extents^14^. They are generally similar to peridotite sampled elsewhere along ridges, considered to be residues of various extents of sub-ridge melting^15^. The mineral chemistry data used here (Table 1) are from previous studies^8,9,11^.

**Table 1:** H_2_O contents in cpx and opx from VLS abyssal peridotites. H_2_O concentrations are measured by infrared spectroscopy. Melting parameters are estimated from published data^9,11^.

Sample	Age*	H_2_O (ppm)		Spinel	Opx	Cpx	^$^F%	Ce (cpx)	Nd (cpx)
	(Ma)	Opx	Cpx	Cr#	Cr#	Cr#		ppm	ppm
S2221-04	9.70	258	487	25.97	11.95	14.12	11	0.072	0.189
S2221-05	9.70	274	898	26.20	12.15	13.04	11		0.065
S1927-02	12.20	383	1005	24.10	10.79	13.85	10		0.091
S1925-71	12.50	237	626	23.90	11.17	13.22	10	0.045	0.145
S1925-75	12.50		615	31.50	12.08	15.28	12	0.037	0.285
S1924-19	12.80	201	529	17.90	8.85	9.83	7	0.030	0.400
S1923-45	13.11		660	26.80	11.56	14.41	11	0.010	0.045
S1923-46	13.11	335	944	25.40	11.22	15.16	10	0.020	0.110
S1924-01	12.80	163	517	16.92	8.15	9.96	6	0.145	0.580
VE1-1	13.90	153	489	18.60	10.06	11.23	7	0.372	1.024
EW9305-15-23	14.07	305	1072	21.40	10.69	11.41	9	0.011	0.085
EW9305-16-1	14.20	210	809	23.40	11.90	13.41	9	0.016	0.119
EW9305-17-5	14.50	216	826	32.60	13.63	12.53	13	0.005	0.083
S1904-76	16.10	304	1008	26.00	12.11	13.21	11	0.006	0.064
S1904-77	16.10	220	629	25.80	11.89	13.13	10	0.004	0.043
S1912-05	18.47	140	407	17.70	8.95	10.94	7	0.005	0.150
S1913-03	18.70	119	475	15.80	9.72	11.32	6	0.036	0.635
S1913-36	18.70		552	21.10	10.21	12.90	8	0.103	0.747

Note: Opx, orthopyroxenes; Cpx, clinopyroxenes and Sp, spinels.

^*^Crustal ages inferred from location, plate boundary geometry and spreading rates^9,11^.

^$^Extent of melting *F* estimated using sp Cr# according to ref. ^19^.

## Results

**Figure 1:**
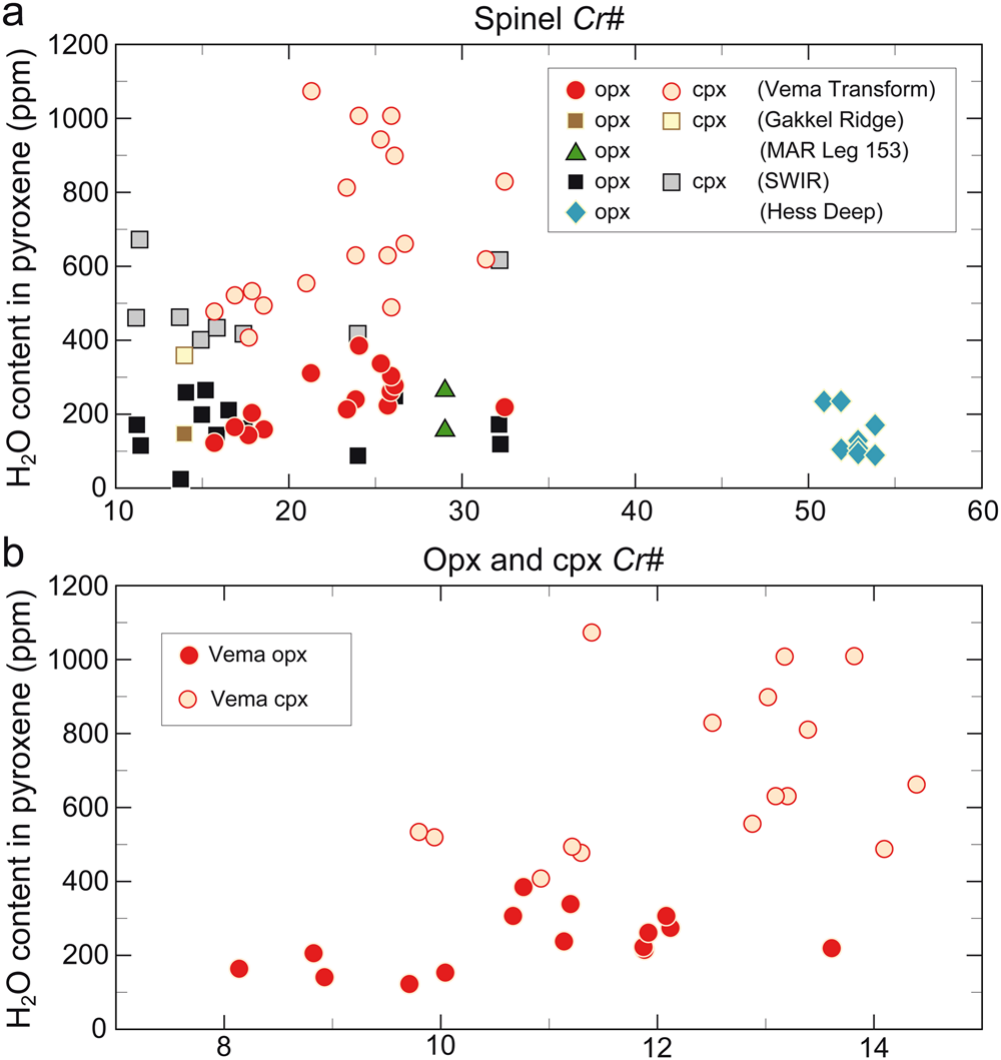
Pyroxene H_2_O content vs extent of melting. (**a**) Variations of H_2_O content vs sp Cr# in opx and cpx from abyssal peridotites from the VLS (opx, red and cpx, orange filled circles), from ODP-Leg 153 boreholes along the Mid Atlantic Ridge^21^ (opx, green triangles), from Hess Deep^27^ (opx, dark cyan diamonds), from Southwest Indian Ridge^22,28^ (opx, black and cpx, gray filled square) and from Gakkel Ridge^22^ (opx, brown and cpx, yellow filled square). Data are listed in Supplementary Table 2. (**b**) H_2_O content in opx (red circle) and in cpx (orange circle) from the VLS peridotites vs opx Cr# and cpx Cr#, respectively.

**Figure 2:**
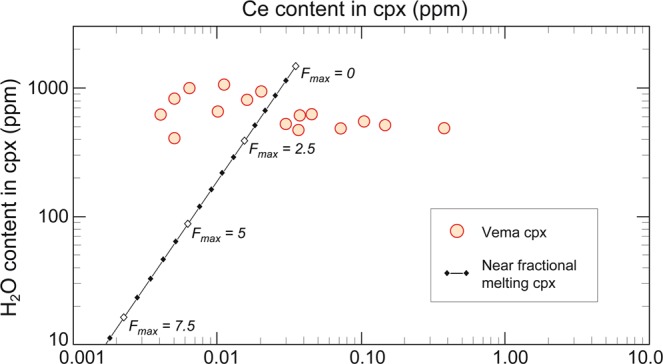
H_2_O variability vs Ce content in cpx from VLS peridotites (orange filled circles). Black solid line indicates co-variation of H_2_O and Ce concentrations at increasing maximum degree of melting *F*_*max*_. Diamonds mark *F*_*max*_ steps of 0.5. The lack of correlation between H_2_O and Ce suggests post-melting H_2_O enrichment in the VLS residual peridotites.

We used infrared spectroscopy to measure the concentration of water or, more precisely, of hydroxyl (OH) defects, in both cpx and opx (see Methods). All measured pyroxene grains display several obvious absorption bands between 2800 and 3700 cm^−1^ (Supplementary Fig. 1) characteristic of the OH-stretching vibration regions in cpx and opx reported in earlier studies^16–18^. The H_2_O content of VLS peridotites ranges from 407 to 1072 ppm in cpx and 119 to 383 ppm in opx (Table 1) and shows negligible core-rim variability (Supplementary Fig. 2). The H_2_O content of both cpx and opx in our samples displays weak positive or no correlation with the spinel chromium number (Cr# = 100Cr/(Al + Cr), in mole fraction) (Fig. 1a), a robust index reflecting degrees of melting of the host peridotites^19^. Similar results were obtained also between H_2_O content and other melting indices, such as Cr in pyroxene (Fig. 1b). These results are surprising because it is experimentally established and naturally observed that H_2_O behaves incompatibly during mantle melting, with a partition coefficient similar to that of Ce^19,20^; therefore, the H_2_O content in melting residues is expected to decrease with degree of melting, contrary to our results. In fact, the concentration of Ce (Table 1) in the VLS peridotite pyroxenes anticorrelates with their degree of melting^9^, in line with Ce being incompatible but in contrast with the behaviour of H_2_O (Fig. 2).

## Discussion

**Figure 3:**
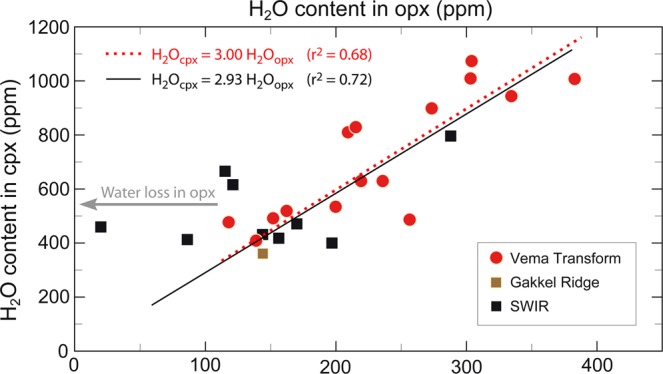
Co-variations of H_2_O contents in cpx and opx from residual peridotites from the VLS (red filled circles), Southwest Indian Ridge^22^ (black filled square) and Gakkel Ridge^22^ (brown filled square). The dotted red line marks regression line evaluated with data from the VLS only, while the black solid line indicates a regression line including all the samples (Supplementary Table 2).

In order to explain these results, we explored two alternative possibilities. One, the correlation H_2_O content-degree of melting in our peridotites is due to processes occurring in the sub-ridge mantle during melting. Two, it is due to processes taking place after melting. The first alternative implies a number of assumptions. One is that the pyroxene H_2_O content is not modified during post melting uplift of the mantle peridotites. This assumption has been shown to be valid in ODP Leg 153 peridotites drilled from the Mid Atlantic Ridge near 23°N, where opx contain H_2_O in the 159-270 ppm range^21^. Also favouring this assumption is our observation that H_2_O is distributed homogeneously within individual opx grains; core-rim profiles show no obvious H_2_O content variations, suggesting no H diffusive loss or addition (Supplementary Fig. 2). Moreover, cpx and opx H_2_O contents are positively correlated with a partition coefficient of 3.0 (Fig. 3), a value close to an average value of 2.6 obtained for pyroxenes in peridotites from oceanic ridges and xenoliths^22^. However, if the pyroxene H_2_O content of the Vema mantle-derived peridotites were due to sub-ridge melting processes, we would expect elements as incompatible as H_2_O, i.e., Ce, Nd, Yb, etc., to behave like H_2_O during melting. Their concentration in pyroxenes would then correlate positively with the concentration of H_2_O. This is clearly not the case, as shown for instance by a plot of Nd versus H_2_O in the Vema cpx (Supplementary Fig. 3). Ce should also follow H_2_O and be higher in the depleted pyroxenes (Fig. 2). However, Ce in contrast to H_2_O, is lower in the depleted pyroxenes^9^.

The behaviour of H_2_O, different from that of elements with similar partition coefficients, is hard to reconcile with the distribution of H_2_O in the Vema mantle peridotites being due solely to partial melting processes. Given the degrees of melting of the Vema peridotites estimated from Cr# of spinel and pyroxenes, and given the experimentally determined partition coefficient^20,23^ D melt-peridotite H_2_O of 0.006 to 0.012, the pre-melting H_2_O content of the mantle source would have to rise up to ~1,500 ppm, an unreasonably high value. Moreover, H_2_O behaves incompatibly during mantle melting, with a partition coefficient similar to that of Cerium^24^. According to the Cr# of spinel, the degree of melting of the VLS peridotites ranges from 6 to 13% (Table 1). A simple calculation predicts almost no water (less than 1 ppm) remaining in abyssal peridotites after a small (>4%) degree of fractional melting, assuming pre-melting sources^25^ with 200 ppm H_2_O and a ${D}_{{H}_{2}O}^{peridotite-melt}$ of 0.008. These additional arguments suggest that the observed water enrichment reflects post-melting hydration in the mantle.

Among post-melting processes we consider first serpentinization and contact metamorphism. The influence of serpentinization is unlikely because hydrogen diffuses sluggishly into pyroxene grains at low temperature (<300 °C) and pressure^26–28^. Serpentinization of the Vema peridotites at the depth of <4 km, occurred mostly near ridge axis^14^ at temperatures <250 °C. Our measured δ^18^O values ([δ^18^O = (^18^O/^16^O)/0.0020052-1]*1000, see Methods), range from 5.81 to 6.09‰ in opx and from 5.52 to 5.82‰ in cpx (Supplementary Table 1). Oxygen isotope fractionations between opx and cpx (D_opx-cpx_ = δ^18^O_opx_-δ^18^O_cpx_) range from 0.17 to 0.35, suggesting oxygen isotope equilibrium at mantle conditions^29^ and excluding serpentinization as a cause of the high H_2_O content of the VLS pyroxenes (Supplementary Fig. 4). Accordingly, measured FTIR patterns confirm the absence of exotic H_2_O in mineral alterations and fluid inclusions and show only the contribution of structural H_2_O (Supplementary Figs. 1 and 2).

Low-pressure contact metamorphism can occur in the lower oceanic crust where discontinuous magma chambers are surrounded by mantle rocks. Degassing from a magma chamber can potentially refill depleted rocks by diffusion in the aureola zone. Aggregated melts differentiating in a magma chamber are characterized by high δ^18^O values; such a process would therefore produce a positive correlation between water content and δ^18^O. This is clearly not the case in the Vema peridotites, as shown in Supplementary Fig. 5.

Experimental studies of the influence of water on melting and phase assemblages in the upper mantle have shown a water content of ~200 ppm in residual nominally anhydrous minerals after incipient melting of lherzolite at the vapour-saturated solidus with pressure ranging from 2.5 to 4 GPa^30–32^. This raises the possibility that some of the investigated upper mantle peridotites may represent parcels of the upper mantle that did not go through significant melting; in fact, the maximum water contents in both pyroxenes of Vema mantle-derived peridotites resemble closely those found in these studies (~300 and 900 ppm for opx and cpx, respectively). However, mantle rocks exposed along the VLS formed originally towards the northern edge of the 80-km-long eastern MAR segment from the sub-axial mantle column, which inevitably went through the melting region. Given the divergent upwelling flow of the solid mantle beneath a ridge segment, it is unlikely that off-axis mantle rocks after incipient melting may converge towards ridge axis.

One possible H_2_O-rich source for post-melting enrichment of the subridge rising mantle might be H_2_O-rich, low degree melts originating by off-axis incipient melting. The production of these low degree melts at the edges of the sub-ridge melting region has been hinted at by several studies^21,22,27,28,30–33^. These small quantities of H_2_O-rich incipient melt may be channelled toward the axis along the sloping base of the thermal lithosphere. Distal melts trapped in the lithosphere-asthenosphere boundary may carry amounts of H_2_O comparable to those we estimated in equilibrium with Vema residual pyroxenes: incipient melts from depleted upper mantle with ~200 ppm H_2_O would contain about 2-3 wt% H_2_O^24,34^.

**Figure 4:**
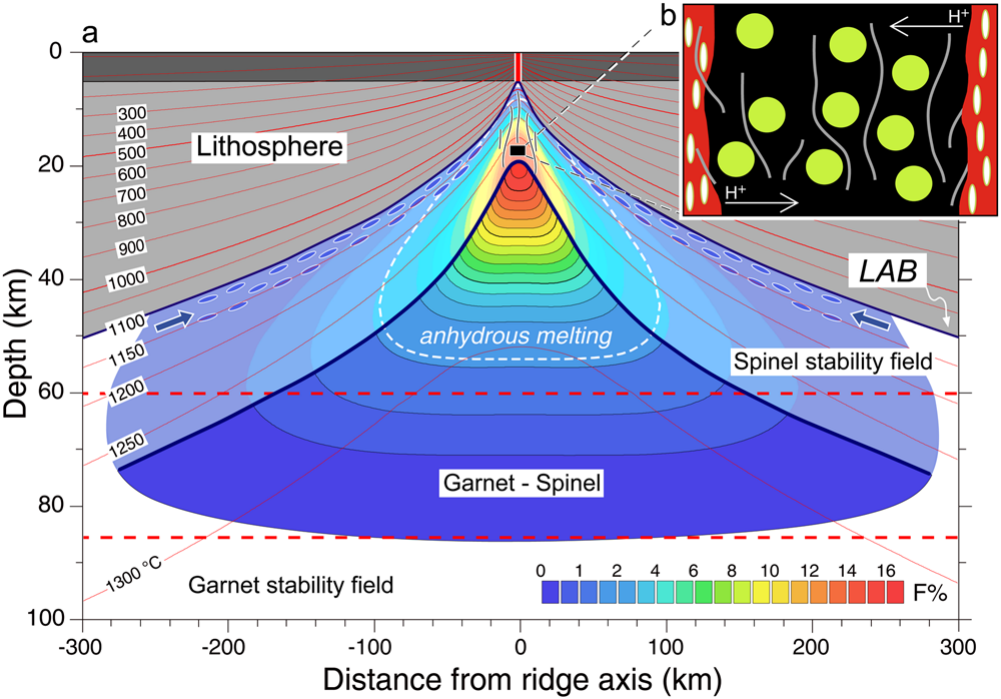
(**a)** Fraction of melt (*F%*, colour coded) generated across the ridge segment in proximity (20 km) to the eastern Vema ridge-transform intersection. Model calculations, following method outlined by refs. ^42,64^, include the effect of water on the peridotite solidus. Mantle temperatures estimated by solving the steady-state advection-diffusion heat equation assuming 0 °C at the seafloor and 1350 °C at 150 km depth, using a three-dimensional domain of mantle flow calculations, with variable grid spacing (512 × 256 × 101), and highest grid resolution at the plate boundaries. Mantle flow velocities were estimated assuming steady-state plate-thickening passive flow^64^ beneath a ridge-transform-ridge plate boundary simulating the Vema transform geometry. Red thick dashed lines indicate boundaries between garnet and spinel stability fields. Mineral proportions in the transition zone between 85 and 60 km are assumed to vary linearly from pure garnet peridotite to pure spinel peridotite^25^. Isotherms are indicated by thin red lines. White thick dashed line marks the region of anhydrous melting, i.e., the sub-region where water is completely exhausted from peridotite nominally anhydrous minerals^25^. Solid thick blue line marks the upper boundary of the region that contributes to melt production (full rainbow scale), i.e. where production rate is positive. The lighter rainbow scale area marks the mantle region where a parcel of melt with a given degree of melting freezes if not extracted from the melting region, i.e., where production rate is negative. The 1100 °C isotherm is assumed to approximate the lithosphere-asthenosphere boundary layer (LAB)^65^. Water-rich low degree melts, produced at the edge of the sub-ridge melting region, percolate at the base of the lithosphere (blue ellipses) where they migrate towards ridge axis. (**b**) Cartoon showing details of the high temperature post-melting region affected by melt flow through dunitic conduits where hydrogen may diffuse from H-rich to H-poor zones of the surrounding upper mantle. Green circles: residual peridotite minerals. Red filled vertical regions: dunitic channels.

Oceanic peridotites, representing fragments of the uppermost zone of the subaxial melting column, show often vein lithologies suggesting interaction with melts^35–37^. We explore the hypothesis that the H_2_O rich melts recorded by our Vema residual peridotites are fractions of distal low-degree melts that migrated along lithosphere-asthenosphere boundary channels towards ridge axis (Fig. 4). These low density/low viscosity melts may tend to accumulate toward the top of the sub-ridge melting column and to react with residual peridotites before dispersing within the low-H_2_O aggregate melts in sub-axial dunite channels.

**Figure 5:**
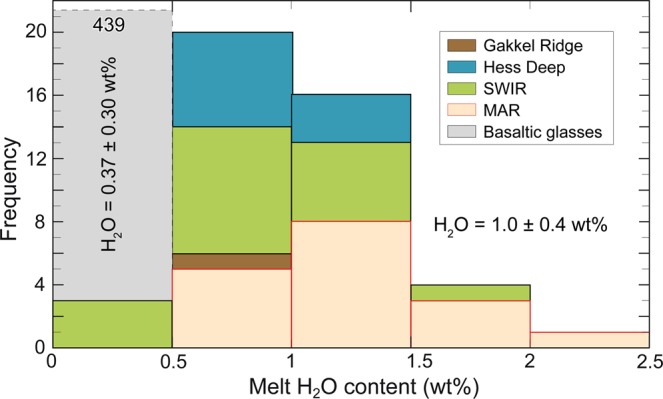
Frequency histogram of predicted H_2_O content of melts assumed in equilibrium with opx from mid ocean ridge abyssal peridotites (Supplementary Table 2). Melts global water estimates average ~1.0 $\pm \,0.4$ wt%. H_2_O contents of 439 basaltic-glasses sampled along the global mid-ocean ridge system are from the PetDB Database^66^ with an average water content of ~0.37 $\pm \,0.3$ wt%.

The H_2_O content of peridotite pyroxenes may help estimate the H_2_O content of the interacting melts, thanks to the experimentally established hydrogen partition coefficients between olivine and pyroxenes $({D}_{{H}_{2}O}^{ol-opx}=0.11\pm 0.01,$ ${D}_{{H}_{2}O}^{ol-cpx}=0.08\pm 0.01)$ and between minerals and basaltic melts $({D}_{{H}_{2}O}^{ol-melt}=0.017\pm 0.0005,\,{D}_{{H}_{2}O}^{opx-melt}=$ $0.019\pm 0.004,\,{D}_{{H}_{2}O}^{cpx-melt}=0.023\pm 0.005)$ at upper mantle pressure (1-3 GPa) and temperature (1230–1380 °C)^38,39^. In fact, the amount of water in olivine may be determined from the water content of coexisting pyroxenes using mineral-mineral partition coefficients and the bulk water concentration for peridotite may be then estimated using mineral modes^22^ (see Methods). Accordingly, the melt H_2_O content retrieved from pyroxenes H_2_O contents may range from 1.0 to 2.5 wt% (average 1.6 ± 0.4 wt%) when olivine H_2_O content is estimated from opx, and from 1.0 to 3.0 wt% (average 1.8 ± 0.6 wt%) when estimated from cpx (Table 2). The differences between melt water contents estimated from opx and from cpx (Table 2) may be due to: (*i*) opx and/or cpx in our peridotites are not in equilibrium with the melt despite the observed equilibrium partitioning of H_2_O between opx and cpx (Fig. 3); (*ii*) the equilibrium partition coefficients of H_2_O between pyroxenes and melt determined experimentally are not appropriate because post-melting processes were not included in the experiments. In fact, experimentally determined ${D}_{{H}_{2}O}^{cpx-opx}$ partition coefficients for peridotite at pressures from 1 to 3 GPa range^40^ from 1.2 to 2.0, with an average of 1.5, although abyssal peridotites display higher values^41^ with an average of 2.2. Given that the experimentally determined mineral/mineral ${D}_{{H}_{2}O}^{cpx-opx}\,$ partition coefficient^38,39^ of 1.4 is about half the value recorded by our samples, we can assume as lower and upper limits the melt H_2_O content estimated from opx and cpx (see Methods). Such high H_2_O melts are unlikely to form by “normal” subridge high-degree partial melting; they may be generated only by very low degrees of melting ranging from ~0.1% to ~0.9%, assuming equilibrium hydrous melting^42^ and 200 ppm H_2_O in the mantle source. These low amounts of incipient melts reflect a low interconnectivity between solid matrix grains and thus, a low attitude to melt migration. A simple compilation of global data indicates that, although never reaching the extreme Vema enrichments, excess water contents are common in “residual” mantle rocks worldwide (Fig. 5 and Supplementary Table 2). Global water contents estimated for equilibrium melts statistically assembled by ridge segments (Fig. 5) do not match any MOR basalts nor mineral-hosted melt inclusions^5,25^. In general, melt H_2_O contents increase with increasing degree of fractionation (decreasing MgO)^43^; an alternative explanation could be that conductive heat flux from the surface causes cooling along the flanks of the melting region. As a consequence, most of the low degree melt produced in the distal parts of the H_2_O rich melting region migrating towards the lithosphere-asthenosphere boundary (LAB) crystalizes and fractionates, increasing the melt H_2_O content and decreasing the melt freezing point^43,44^.

**Table 2:** H_2_O contents in olivine(ol) are estimated from opx and cpx water contents adopting mineral-mineral partition coefficients ${D}_{{H}_{2}O}^{ol-opx}=0.11\,$ and ${D}_{{H}_{2}O}^{ol-cpx}=0.08$ from refs. ^38,39^.

*Sample*	*C*_*H2O*_^*ol(opx)*^	*C*_*H2O*_^*ol(cpx)*^	*C*_*H2O*_^*melt(opx)*^	*C*_*H2O*_^*melt(cpx)*^	*ol*	*opx*	*cpx*	*sp*	*D*_*H2O*_^*bulk-melt*^	*C*_*H2O*_^*bulk(a)*^	*C*_*H2O*_^*bulk(b)*^	*C*_*H2O*_^*melt(a)*^	*C*_*H2O*_^*melt(b)*^
	*(ppm)*	*(ppm)*	*(wt%)*	*(wt%)*						*(ppm)*	*(ppm)*	*(wt%)*	*(wt%)*
S2221-04	28	39	1.4	2.1	0.75	0.20	0.03	0.02	0.0058	87	95	1.5	1.7
S2221-05	30	72	1.4	3.9	0.75	0.22	0.02	0.01	0.0059	102	134	1.7	2.3
S1927-02	42	80	**2.0**	4.4	0.76	0.18	0.04	0.02	0.0058	145	174	**2.5**	**3.0**
S1925-71	26	50	1.3	2.7	0.73	0.21	0.05	0.02	0.0062	97	114	1.5	1.8
S1925-75		49		2.7	0.76	0.17	0.05	0.02	0.0057		69		1.2
S1924-19	22	42	1.1	2.3	0.74	0.19	0.05	0.02	0.0059	79	94	1.3	1.6
S1923-45		53		2.9	0.70	0.24	0.05	0.01	0.0069		69		**1.0**
S1923-46	37	76	1.8	4.1	0.71	0.24	0.04	0.01	0.0067	143	170	2.1	2.5
S1924-01	18	41	0.9	2.3	0.76	0.15	0.07	0.02	0.0058	76	93	1.3	1.6
VE1-1	17	39	0.8	2.1	0.77	0.17	0.04	0.02	0.0055	60	77	1.1	1.4
EW9305-15-23	34	86	1.6	**4.7**	0.73	0.21	0.05	0.02	0.0063	137	175	2.2	2.8
EW9305-16-1	23	65	1.1	3.5	0.74	0.21	0.04	0.02	0.0061	94	124	1.5	2.0
EW9305-17-5	24	66	1.1	3.6	0.71	0.24	0.04	0.01	0.0066	102	132	1.5	2.0
S1904-76	33	81	1.6	4.4	0.69	0.25	0.05	0.01	0.0070	145	178	2.1	2.5
S1904-77	24	50	1.2	2.7	0.70	0.25	0.04	0.01	0.0068	98	116	1.1	1.7
S1912-05	15	33	0.7	**1.8**	0.77	0.16	0.05	0.02	0.0055	55	68	1.0	1.2
S1913-03	13	38	**0.6**	2.1	0.75	0.16	0.06	0.02	0.0058	58	77	**1.0**	1.3
S1913-36		44		2.4	0.75	0.18	0.05	0.02	0.0059		60		1.0

Mineral modes are estimated following methods of refs. ^9,59^. Predicted melt water contents are calculated from opx, cpx and bulk rock water contents adopting partition coefficients ${D}_{{H}_{2}O}^{ol-melt}=0.0017$, ${D}_{{H}_{2}O}^{opx-melt}=0.019\,$ and ${D}_{{H}_{2}O}^{cpx-melt}=0.023\,$ from refs. ^38,39^ and assuming melt in equilibrium with residual peridotites (see Methods).

Next we will investigate the role of post-melting metasomatism and re-equilibration of water in the nominally anhydrous mantle rocks minerals, assuming that H_2_O-rich incipient melts may be extracted and focused toward ridge axis. Refractory peridotite residues may be more susceptible to shallow mantle metasomatism than fertile lherzolites^45^. Therefore, the slight positive correlation between H_2_O content and degree of melting may simply reflect post melting metasomatic enrichment of incompatible elements, including H_2_O, in the more refractory peridotite. Shallow mantle metasomatism should mobilize not only H_2_O, but also LREE. However, the VLS peridotites are characterized by cpx with LREE-depleted patterns^9^ and spinel with less than 0.1 wt% TiO_2_, indicating weak or no mantle metasomatism^9^. Modelling based on REE and Ti-Zr in residual cpx also indicates weak re-fertilization of the residual source by small (~0.2%) amounts of partially aggregated melt^9^. If re-fertilization was the main cause, we would expect the peridotite H_2_O content to correlate positively with chemical indices reflecting the extent of metasomatic/melt-rock reactions such as Ce/Yb, and Na_2_O in cpx (Supplementary Fig. 3). Absence of these correlations suggests refertilization is not the main cause of H_2_O enrichment in the VLS peridotites (Supplementary Fig. 6).

Hydrogen diffusion into pyroxenes at mantle depths after cessation of sub ridge partial melting might have occurred in East Pacific Rise^27^ (Hess Deep) and Mid Atlantic Ridge^21,46,47^ peridotites. H diffusion in and out of pyroxenes is favoured by high temperature. The Vema mantle peridotites have risen slowly from mantle depths after melting has stopped roughly 20 km below the seafloor (Fig. 4). The rate of ascent is similar to the half spreading rate^8^, i.e., ~15 mm/yr: the ascent within the mantle up to the base of the sub ridge lithosphere (~5 km below seafloor) lasts roughly 1 Ma. A good interval of this upward motion will take place at temperatures ranging from ~1250 °C to ~900 °C. During ascent of the peridotite, melts may flow in dunitic conduits and veins cross cutting the uprising mantle. The spacing of these conduits could be quite narrow^48^ for a nearly continuous melt extraction as we observe at the VLS. Given its high diffusion coefficient, H could diffuse from H-rich to H-depleted zones in the surrounding mantle^49^. The diffusion coefficient *D* of H in pyroxene within the predicted temperature range will vary from ~1 × 10^−10^ to ~1 × 10^−12^ m^2^/s, while REE diffusion coefficients are of the order of 1 × 10^−21^ m^2^/s, that is, orders of magnitude lower. Thus, in 1 Ma hydrogen could travel as far as ~11 ÷ 112 m (diffusion length scale = 2*√D t), while a REE would be able to travel only ~0.35 mm. This process would explain why H is decoupled from other incompatible elements in our peridotites (Supplementary Figs. 3 and 7). Higher hydrogen diffusion coefficients in cpx than that in opx would also explain why hydrogen has been mostly taken up by cpx rather than by opx, since mineral-mineral (cpx-opx) water partition coefficients recorded by our data are about twice those determined experimentally (Fig. 3). However, the hydrogen diffusion coefficients in natural opx reported in literature^50^ are of the same order of magnitude as hydrogen diffusion coefficients for diopside^51^, although few experiments are available for natural orthopyroxenes, with total absence of hydrogen diffusion data for pure enstatite^52^.

Diffusion of hydrogen was already suggested to explain low variability of water content relative to Ce in nominally anhydrous mantle minerals from Colorado Plateau peridotite xenoliths^49^. However, hydrogen diffusion requires a prolonged post-melting residence time of mantle rocks at mantle conditions. The negative correlation between opx water contents and mantle equilibration temperatures observed comparing peridotites from ODP Legs 153 and 149 supports this idea^46,47^, i.e., lower equilibration temperatures imply longer H diffusion times during mantle rise and consequently higher water contents. Oxygen isotopes tell here the same story. In fact, δ^18^O and water content for opx and cpx define a slight negative trend (Supplementary Fig. 5) suggesting that at nominally zero W/R ratio, high temperature H_2_O diffusion may have produced a slight depletion in oxygen isotope composition. High temperature H_2_O incorporation occurs upon cooling according to the oxygen isotopic equilibrium fractionation in opx and cpx, that in turn depends on equilibration temperatures. This explains why opx and cpx oxygen isotope contents do not correlate positively with their H_2_O contents.

Our post-melting models of H_2_O enrichment of sub-ridge mantle peridotites do not exclude that different parcels of pre-melting mantle along the VLS may have contained different amounts of H_2_O, and that these different H_2_O contents, in addition to temperature, may have caused temporal variations of degree of melting. However, post-melting H diffusion and H_2_O redistribution make it difficult to reconstruct pre-melting H_2_O contents. If our models are correct, it would follow that, due to post melting mobility of H in the suboceanic upper mantle, estimates of the H_2_O content of the pre-melting mantle source of MORB based on the H_2_O content of abyssal peridotite pyroxenes, may be affected by strong uncertainty.

## Methods

### FTIR measurements of H_2_O content

Water content determinations followed methods of ref. ^53^. Infrared measurements were carried out in 10 to 15 relatively large and clean grains of each mineral picked from the peridotites. The selected grains were mounted in a self-supporting epoxy matrix, and double polished to a thickness of ~0.2 mm. We obtained infrared unpolarized spectra at wavelengths ranging from 1250 to 4000 cm^−1^ on a Nicolet 5700 FTIR spectrometer, coupled with a Continuum microscope at USTC, using a KBr beam-splitter and a liquid-nitrogen cooled MCT-A detector. A total of 128 scans were accumulated for each spectrum at a 4 cm^−1^ resolution. The aperture size was set from 30 × 30 μm to 100 × 100 μm, depending on the size and quality of the mineral grains to be analyzed. Accurate determination of OH concentrations in optically anisotropic minerals obtained by non-polarized light on unoriented grains was proven to be a reliable method both theoretically^54^ and practically^55^. The mineral water content was calculated by the transformed Beer-Lambert law:

1 $$C=3A/(I\cdot t),$$

where *C* is the water content of minerals in ppm, *A* is the non-polarised integral absorbance, *I* is the absorption coefficient (7.09 ppm^−1^cm^−2^ for cpx and 14.84 ^−1^cm^−2^ for opx, ref. ^56^) and *t* is the thickness in cm, measured by a digimatic indicator for each grains. The average value was used to obtain water content. Uncertainties in water contents calculated from Eq. (1) derive from: (*i)* using non-polarized infrared beams on non-oriented minerals (<10%); (*ii)* baseline correction (<5%); (*iii)* variable sample thickness (<3%); and (*iv)* differences between the absorption coefficients (<10%) of our samples and those of the samples used by ref. ^57^ due to differences in composition. The total uncertainty is estimated to be less than 20–30%.

### Water content of melt in equilibrium with residual peridotite

The water contents of the percolating melts in equilibrium with the residual peridotites were estimated calculating bulk water concentration of peridotites using mineral modes and determining bulk H_2_O partition coefficient between peridotite and melt using experimentally determined partition coefficients between minerals and melt^58^:

2 $${C}_{{H}_{2}O}^{melt}=\frac{{C}_{{H}_{2}O}^{bulk}}{{D}_{{H}_{2}O}^{bulk-melt}}=\frac{{C}_{{H}_{2}O}^{ol}\,{X}_{ol}+{C}_{{H}_{2}O}^{cpx}\,{X}_{cpx}+{C}_{{H}_{2}O}^{opx}\,{X}_{opx}}{{D}_{{H}_{2}O}^{ol-melt}\,{X}_{ol}+{D}_{{H}_{2}O}^{cpx-melt}\,{X}_{cpx}+{D}_{{H}_{2}O}^{opx-melt}\,{X}_{opx}}=\frac{\sum _{j}{C}_{{H}_{2}O}^{j}\,{X}_{j}}{\sum _{j}{D}_{{H}_{2}O}^{j-melt}\,{X}_{j}},$$

with *j* = *ol, cpx, opx* and where ${C}_{{H}_{2}O}^{melt}$, ${C}_{{H}_{2}O}^{bulk}$ and ${C}_{{H}_{2}O}^{j}$ are the water contents of the melt, of the bulk peridotite and of olivine and pyroxenes; ${D}_{{H}_{2}O}^{bulk-melt}$ and ${D}_{{H}_{2}O}^{j-melt}$ are the partition coefficients of water between bulk peridotite and melt, and between olivine-pyroxenes and melt; and ${X}_{j}$ are the mineral abundances of olivine, cpx and opx, respectively.

The partition coefficients of water between minerals and melt are given by definition:

3 $${D}_{{H}_{2}O}^{j-melt}=\frac{{C}_{{H}_{2}O}^{j}}{{C}_{{H}_{2}O}^{melt}}\,\Rightarrow \,{C}_{{H}_{2}O}^{melt}=\frac{{C}_{{H}_{2}O}^{j}}{{D}_{{H}_{2}O}^{j-melt}}\,\Rightarrow \,{C}_{{H}_{2}O}^{j}={D}_{{H}_{2}O}^{j-melt}\cdot {C}_{{H}_{2}O}^{melt}.$$

Partition coefficients of water between peridotite mineral assemblages and melt determined experimentally may depend from mineral chemistry (i.e., Al_2_O_3_), oxygen fugacity and P-T conditions^22–24,38,39^. Here we assume water partition coefficients determined experimentally at upper mantle pressure (1-3 GPa) and temperature (1230–1380 °C) of ref. ^38^ $(i.e.,\,{D}_{{H}_{2}O}^{ol-melt}=0.017\pm 0.0005,\,{D}_{{H}_{2}O}^{opx-melt}=0.019\pm 0.004,\,{D}_{{H}_{2}O}^{cpx-melt}=0.023\pm 0.005$). Assuming that the mineral phases are in equilibrium with the melt and that the adopted partition coefficients reflect exactly the observed equilibrium, then Eqs. (2) and (3) are equivalent. Taking into account uncertainties in the mineral water content determinations and in the experimental determined partition coefficients, Eq. (3) provides different values of melt water content for each mineral phase of the residual peridotite:

4 $${C}_{{H}_{2}O}^{melt(j)}=\frac{{C}_{{H}_{2}O}^{j}}{{D}_{{H}_{2}O}^{j-melt}}\,\Rightarrow \,{C}_{{H}_{2}O}^{j}={D}_{{H}_{2}O}^{j-melt}\cdot {C}_{{H}_{2}O}^{melt(j)},$$

where ${C}_{{H}_{2}O}^{melt(j)}$ are the melt water contents estimated from mineral phase *j*. By replacing ${C}_{{H}_{2}O}^{j}$ from Eq. (4) into Eq. (2), we obtain:

5 $$\begin{array}{c}{C}_{{H}_{2}O}^{melt}=\frac{{C}_{{H}_{2}O}^{melt(ol)}{D}_{{H}_{2}O}^{ol-melt}{X}_{ol}+{C}_{{H}_{2}O}^{melt(cpx)}{D}_{{H}_{2}O}^{cpx-melt}{X}_{cpx}+{C}_{{H}_{2}O}^{melt(opx)}{D}_{{H}_{2}O}^{opx-melt}{X}_{opx}}{{D}_{{H}_{2}O}^{ol-melt}\,{X}_{ol}+{D}_{{H}_{2}O}^{cpx-melt}\,{X}_{cpx}+{D}_{{H}_{2}O}^{opx-melt}\,{X}_{opx}}=\frac{\sum _{j}{C}_{{H}_{2}O}^{melt(j)}{D}_{{H}_{2}O}^{j-melt}{X}_{j}}{\sum _{j}{D}_{{H}_{2}O}^{j-melt}\,{X}_{j}}\\ \,{C}_{{H}_{2}O}^{melt}=\frac{\sum _{j}{C}_{{H}_{2}O}^{melt(j)}\,{w}_{j}}{\sum _{j}{w}_{j}}\,with\,\,{w}_{j}={D}_{{H}_{2}O}^{j-melt}\,{X}_{j}\,and\,\,\sum _{j}\,{X}_{j}\ne 0\end{array}$$

Equation (5) represents a weighted average of melt water contents estimated from mineral phases of the residual peridotite. The mean-value theorem states that results for ${C}_{{H}_{2}O}^{melt}$ are always in between the minimum and maximum value of the estimated melt water contents (${C}_{{H}_{2}O}^{melt(j)}$) for each mineral phase compositions *X*_*j*_, i.e.:

6 $$\min ({C}_{{H}_{2}O}^{melt(j)})\le {C}_{{H}_{2}O}^{melt}\le \,\max ({C}_{{H}_{2}O}^{melt(j)})\,\,\,\,\,\forall \,{X}_{j}\,.$$

Our samples contain no olivine relicts. Although we were able to estimate mineral abundances in our samples following methods of refs. ^9,59^ (Tab. 2), we were not able to measure any olivine water contents. Thus, olivine water contents have to be inferred from water contents of pyroxenes adopting mineral-mineral partition coefficients, i.e.:

7 $$\{\begin{array}{c}{C}_{{H}_{2}O}^{ol(opx)}={D}_{{H}_{2}O}^{ol-melt}\cdot {C}_{{H}_{2}O}^{melt(opx)}={D}_{{H}_{2}O}^{ol-melt}\cdot \frac{{C}_{{H}_{2}O}^{opx}}{{D}_{{H}_{2}O}^{opx-melt}}={D}_{{H}_{2}O}^{ol-opx}\cdot {C}_{{H}_{2}O}^{opx}\,\,(a)\\ {C}_{{H}_{2}O}^{ol(cpx)}={D}_{{H}_{2}O}^{ol-melt}\cdot {C}_{{H}_{2}O}^{melt(cpx)}={D}_{{H}_{2}O}^{ol-melt}\cdot \frac{{C}_{{H}_{2}O}^{cpx}}{{D}_{{H}_{2}O}^{cpx-melt}}={D}_{{H}_{2}O}^{ol-cpx}\cdot {C}_{{H}_{2}O}^{cpx}\,\,(b)\end{array}$$

where ${C}_{{H}_{2}O}^{ol(opx)}$ and ${C}_{{H}_{2}O}^{ol(cpx)}$ are olivine water contents inferred from opx and cpx water contents; ${D}_{{H}_{2}O}^{ol-opx}$ and ${D}_{{H}_{2}O}^{ol-cpx}$ are mineral-mineral partition coefficients. Here, we adopted those of ref. ^38^, i.e., ${D}_{{H}_{2}O}^{ol-opx}=0.11\pm 0.01,\,{D}_{{H}_{2}O}^{ol-cpx}$. $=0.08\pm 0.01$Replacing Eq. (7) into Eq. (2) we obtain:

8 $$\{\begin{array}{c}{C}_{{H}_{2}O}^{melt(a)}=\frac{{C}_{{H}_{2}O}^{melt(opx)}{D}_{{H}_{2}O}^{ol-melt}{X}_{ol}+{C}_{{H}_{2}O}^{melt(cpx)}{D}_{{H}_{2}O}^{cpx-melt}{X}_{cpx}+{C}_{{H}_{2}O}^{melt(opx)}{D}_{{H}_{2}O}^{opx-melt}{X}_{opx}}{{D}_{{H}_{2}O}^{ol-melt}\,{X}_{ol}+{D}_{{H}_{2}O}^{cpx-melt}\,{X}_{cpx}+{D}_{{H}_{2}O}^{opx-melt}\,{X}_{opx}}\\ {C}_{{H}_{2}O}^{melt(b)}=\frac{{C}_{{H}_{2}O}^{melt(cpx)}{D}_{{H}_{2}O}^{ol-melt}{X}_{ol}+{C}_{{H}_{2}O}^{melt(cpx)}{D}_{{H}_{2}O}^{cpx-melt}{X}_{cpx}+{C}_{{H}_{2}O}^{melt(opx)}{D}_{{H}_{2}O}^{opx-melt}{X}_{opx}}{{D}_{{H}_{2}O}^{ol-melt}\,{X}_{ol}+{D}_{{H}_{2}O}^{cpx-melt}\,{X}_{cpx}+{D}_{{H}_{2}O}^{opx-melt}\,{X}_{opx}}\end{array}.$$

Equation (8) and the mean-value theorem imply that the melt water content predictions ${C}_{{H}_{2}O}^{melt(a)}$ and ${C}_{{H}_{2}O}^{melt(b)}$ satisfy inequality ${C}_{{H}_{2}O}^{melt(opx)}\le {C}_{{H}_{2}O}^{melt(a)}\le {C}_{{H}_{2}O}^{melt(b)}\le {C}_{{H}_{2}O}^{melt(cpx)}$ for each mineral phase abundances (*X*_*j*_). These results suggest that melt water contents estimated from opx and cpx represent the lower and upper limits of water contents of melts in equilibrium with residual peridotites regardless of their mineral abundances.

### Oxygen isotopic ratios

Following method of ref. ^60^, oxygen isotopes were measured at the Consiglio Nazionale delle Ricerche-Istituto di Geoscienze e Georisorse of PISA by laser fluorination^61^, reacting 1 to 1.5 mg opx and cpx fragments in F2 gas^62^. We irradiated the samples with a 25 W CO_2_ laser operating at a wavelength of 10.6 μm (ref. ^63^). Three pre-fluorination steps were made before running new sets of analyses in order to remove the moisture in the holder and in the line. O_2_ produced during laser fluorination together with excess fluorine were passed through potassium chloride salt; excess fluorine was converted into a potassium-fluoride salt and chlorine gas. A cryogenic trap cooled at liquid nitrogen temperature was then used to freeze chlorine. After purification, O_2_ was trapped over a cold finger filled with 5 A zeolites^62^, and transferred to a Finnigan Delta Plus Mass Spectrometer for oxygen isotopic analysis. The international quartz standard NBS 30 and in-house laboratory standard Quartz Merck Standard (QMS) were measured at the beginning of each analytical session. Mineral sequences were started after the standards reached the accepted values: five to six standards were measured during each set of analyses. The average δ^18^O value of NBS30 and QMS is 14.05 ± 0.17‰ (1σ), and 5.24 ± 0.15‰ (1σ), respectively. All δ^18^O values are relative to SMOW (standard mean ocean water, ^18^O/^16^O = 2005.2 × 10^−6^). At least two fragments were analyzed for each mineral, and the variations within the same sample are less than the precision of standards.

## Supplementary information

High H2O Content in Pyroxenes of Residual Mantle Peridotites at a Mid Atlantic Ridge Segment.
