# Supplementary material for: High H2O Content in Pyroxenes of Residual Mantle Peridotites at a Mid Atlantic Ridge Segment
Source: Sci Rep. 2020 Jan 17;10:579. doi: 10.1038/s41598-019-57344-4 (PMC6969183; doi:10.1038/s41598-019-57344-4)
Supplement: Supplementary file 1 — High H2O Content in Pyroxenes of Residual Mantle Peridotites at a Mid Atlantic Ridge Segment. [file 41598_2019_57344_MOESM1_ESM.docx]

## Supplementary Information

High H_2_O Content in Pyroxenes of Residual Mantle Peridotites at a Mid Atlantic Ridge Segment

Pei Li^1*^, Qun-Ke-Xia^1^, Luigi Dallai^2^, Enrico Bonatti^3,4^, Daniele Brunelli^4,5^, Anna Cipriani^3,5^ and Marco Ligi^4^

*^1^ School of Earth Sciences, Zhejiang University, Hangzhou 310027, China*

*^2^ Istituto di Geoscienze e Georisorse - CNR, Via G. Moruzzi 1, 56124, Pisa, Italy*

*^3^ Lamont-Doherty Earth Observatory of Columbia University, Palisades, New York 10964, USA*

*^4^ Istituto di Scienze Marine – CNR, via Gobetti 101, 40129, Bologna, Italy*

*^5^ Dipartimento di Scienze Chimiche e Geologiche, Università di Modena e Reggio Emilia, Modena 41100, Italy*

**This PDF file includes:**

Supplementary Figures 1 to 7

Supplementary Table 1 to 2

References

* To whom correspondence should be addressed. E-mail: *peili@zju.edu.cn*

**
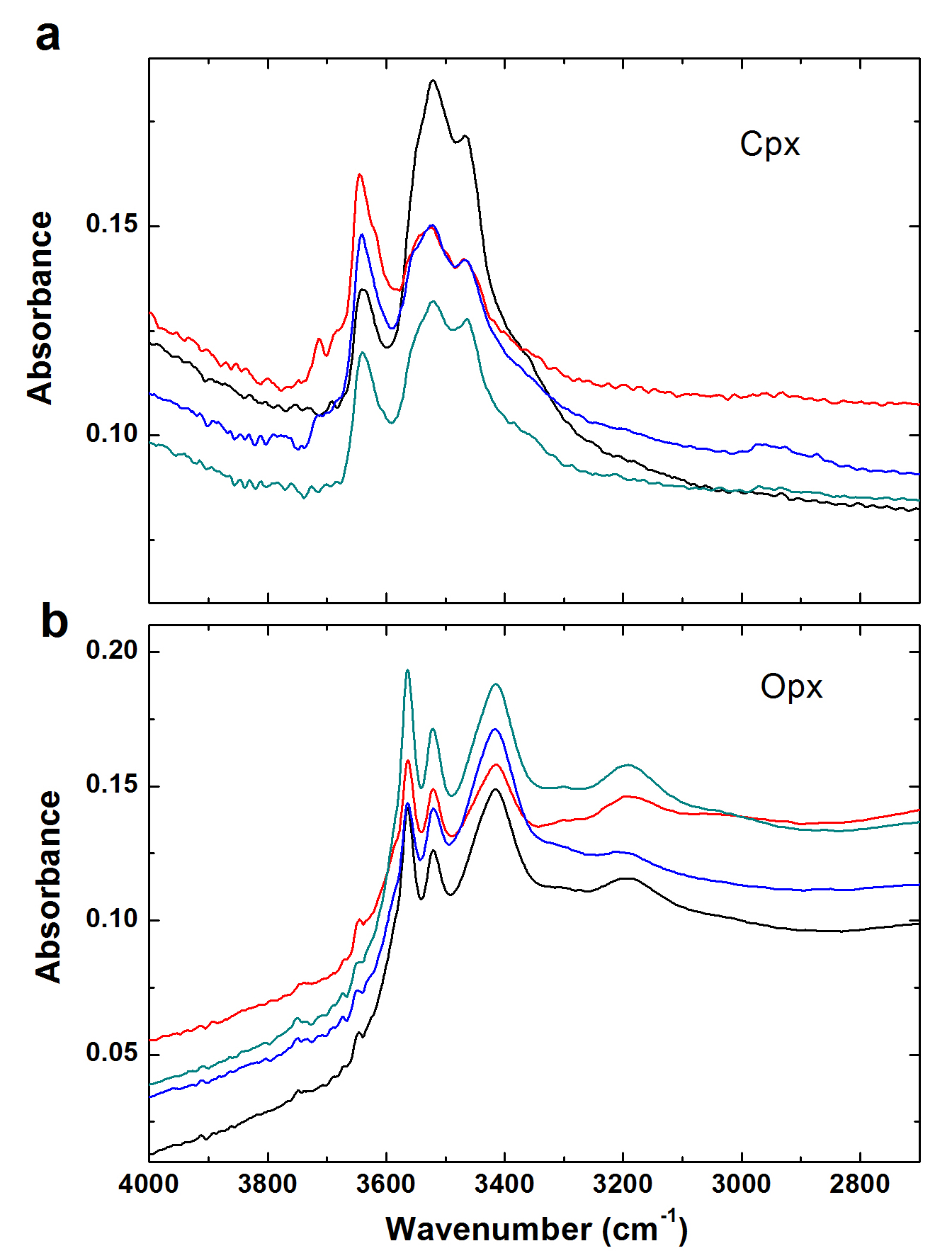
**

**Supplementary Figure 1.** Representative non-polarized IR absorption spectra for mineral phases of VLS abyssal peridotites. **a**, cpx. **b**, opx. The absorbance spectrum in **a** and **b** is normalized to 1 cm thickness. Both spectra were offset vertically for clarity.

**
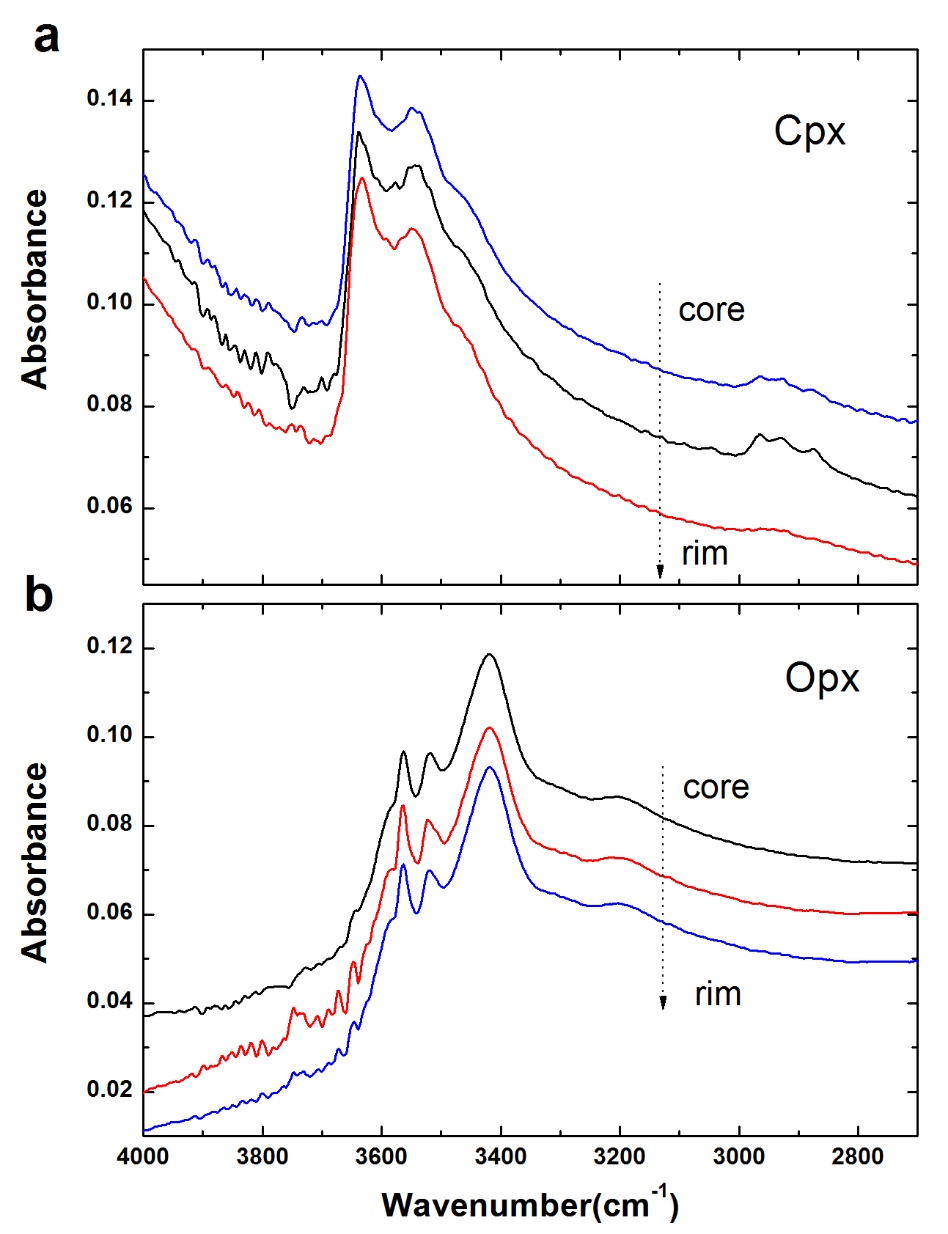
**

**Supplementary Figure 2.** Representative IR profile measurement with absorbance spectra offset vertically for clarity. **a**, cpx. **b**, opx. The thickness of the measured grains is ~ 0.1 mm.

**
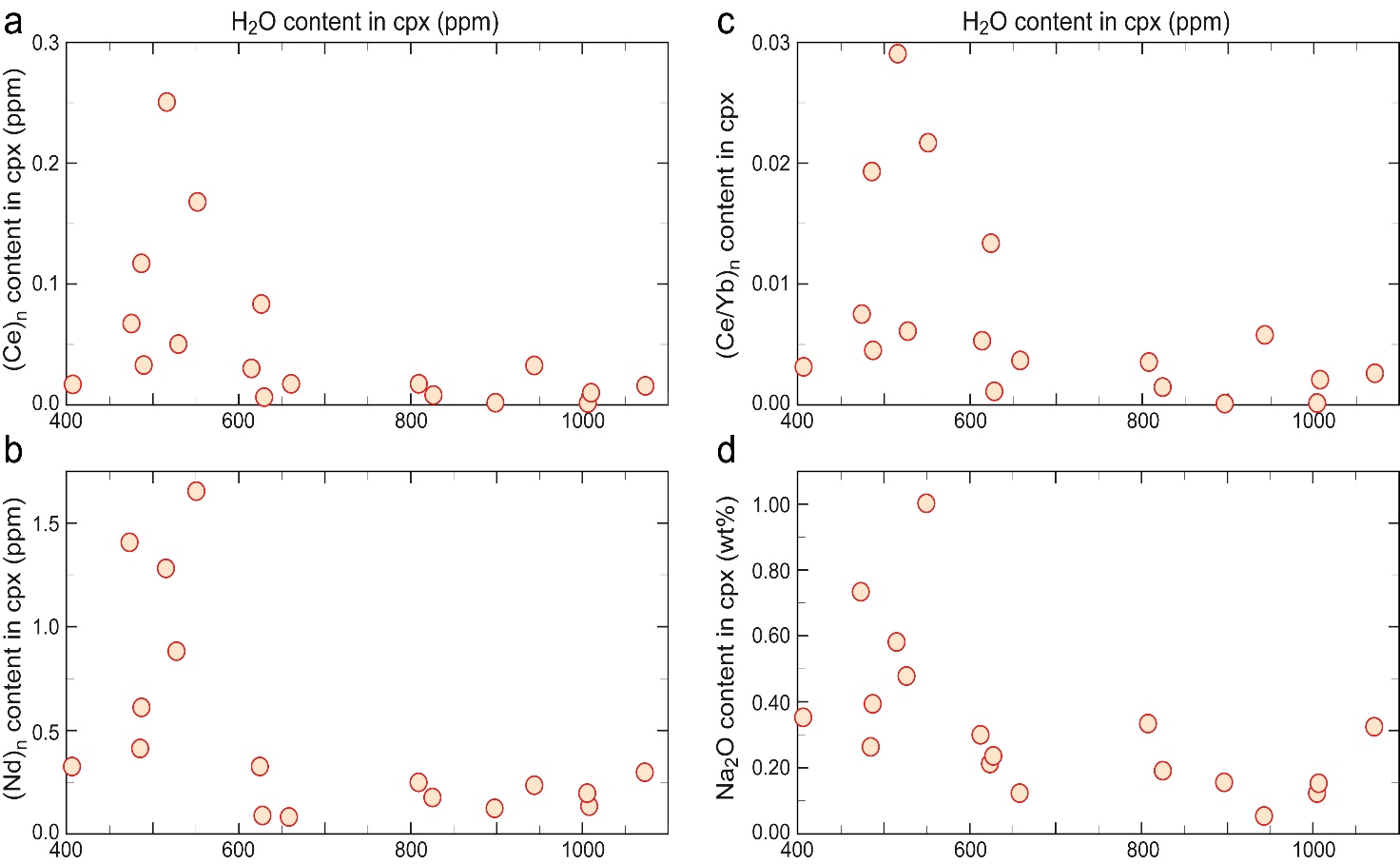
**

**Supplementary Figure 3 |** Variations of H_2_O content in cpx versus chemical indices reflecting the extent of melting and of metasomatic/melt-rock reactions. Chondrite-normalized (Ce)_n_, (Nd)_n_ and (Ce/Yb)_n_ contents were obtained using chondritic values from ref. 69. **a**, (Ce)_n_ in cpx. **b,** (Nd)_n_ in cpx. **c,** (Ce/Yb)_n_ ratio in cpx. **d,** Na_2_O (wt%) in cpx. H_2_O contents do not correlate with other incompatible elements such as Ce, Nd, Yb and Na, that should behave like H_2_O during melting and/or during refertilization by metasomatic/melt-rock reactions. This suggests a non-metasomatic post-melting H_2_O enrichment of VLS abyssal peridotites.

**
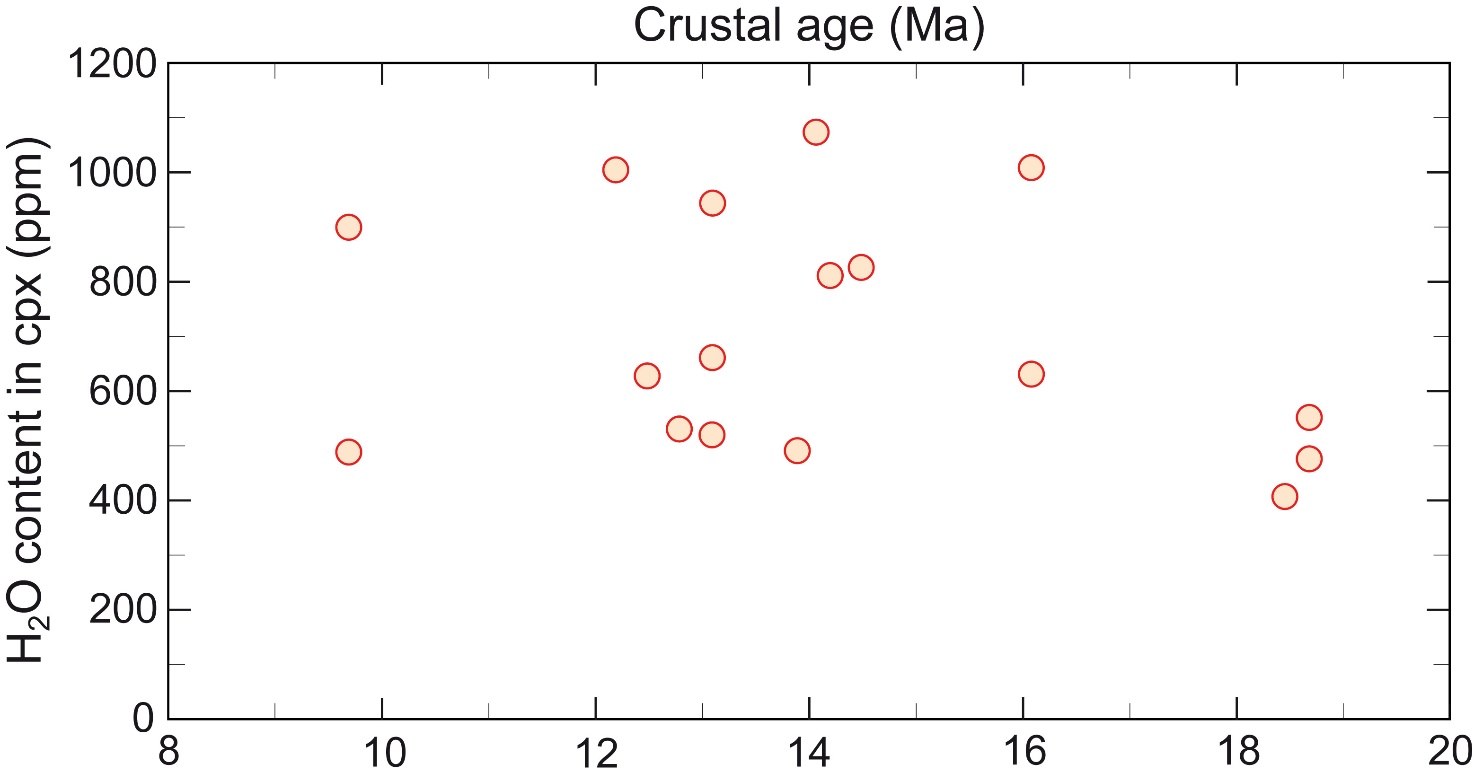
**

**Supplementary Figure 4.** Variations of H_2_O in cpx of VLS residual peridotites versus age of the associated magmatic crust. Residual abyssal peridotites from the VLS have experienced a similar degree of serpentinization with closure temperatures < 250 °C, estimated from stable isotopes that decrease with distance from the ridge axis^14^. δ^18^O data suggest oxygen isotope equilibrium at mantle conditions, excluding serpentinization as a cause of the high H_2_O content of the VLS pyroxenes.

**
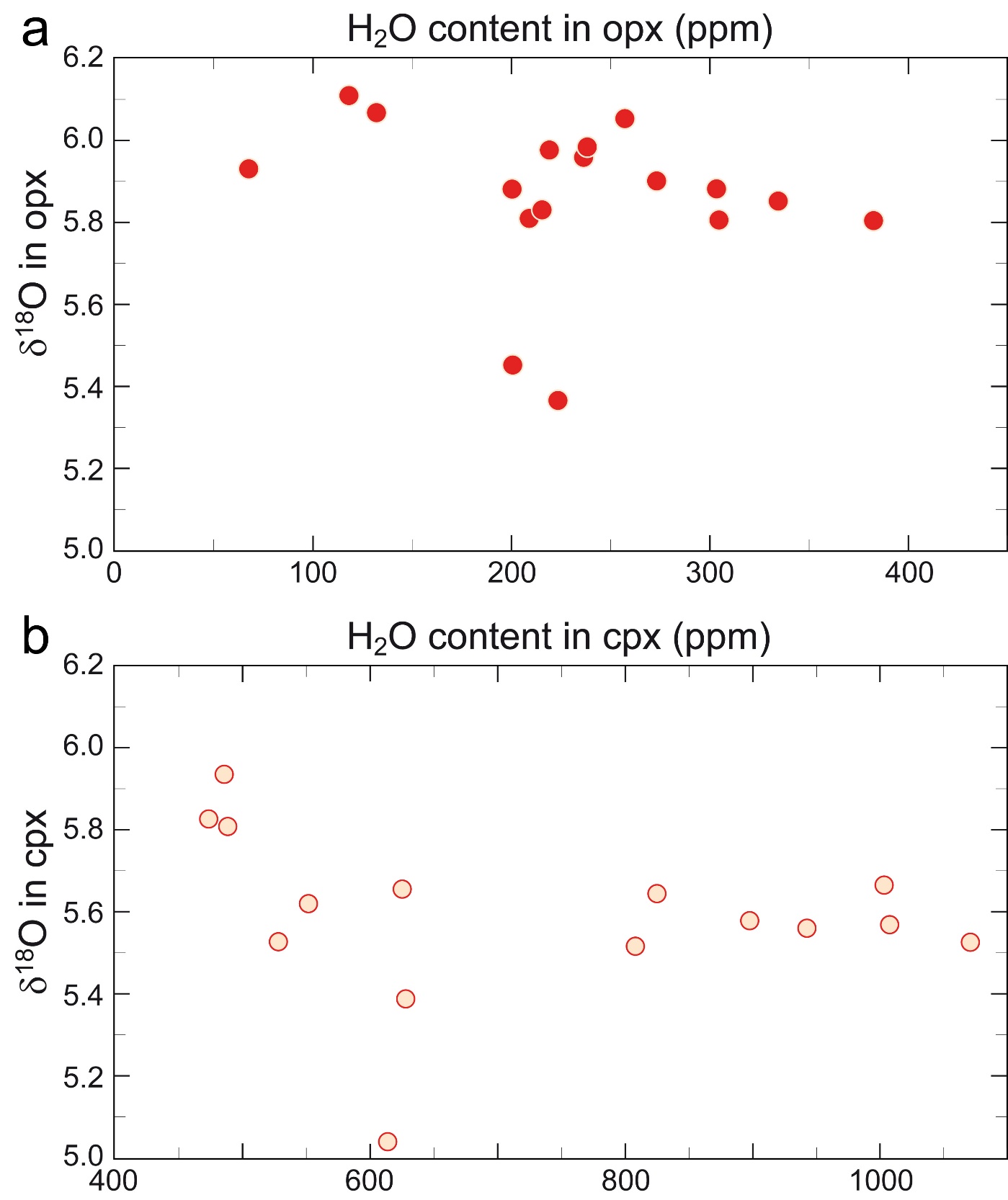
**

**Supplementary Figure 5.** Oxygen isotopic ratio (δ^18^O) variations versus H_2_O contents in VLS residual abyssal peridotites. **a,** δ^18^O vs H_2_O content in opx. **b,** δ^18^O vs H_2_O content in cpx. Oxygen isotopic data are shown in Supplementary Table 1

**
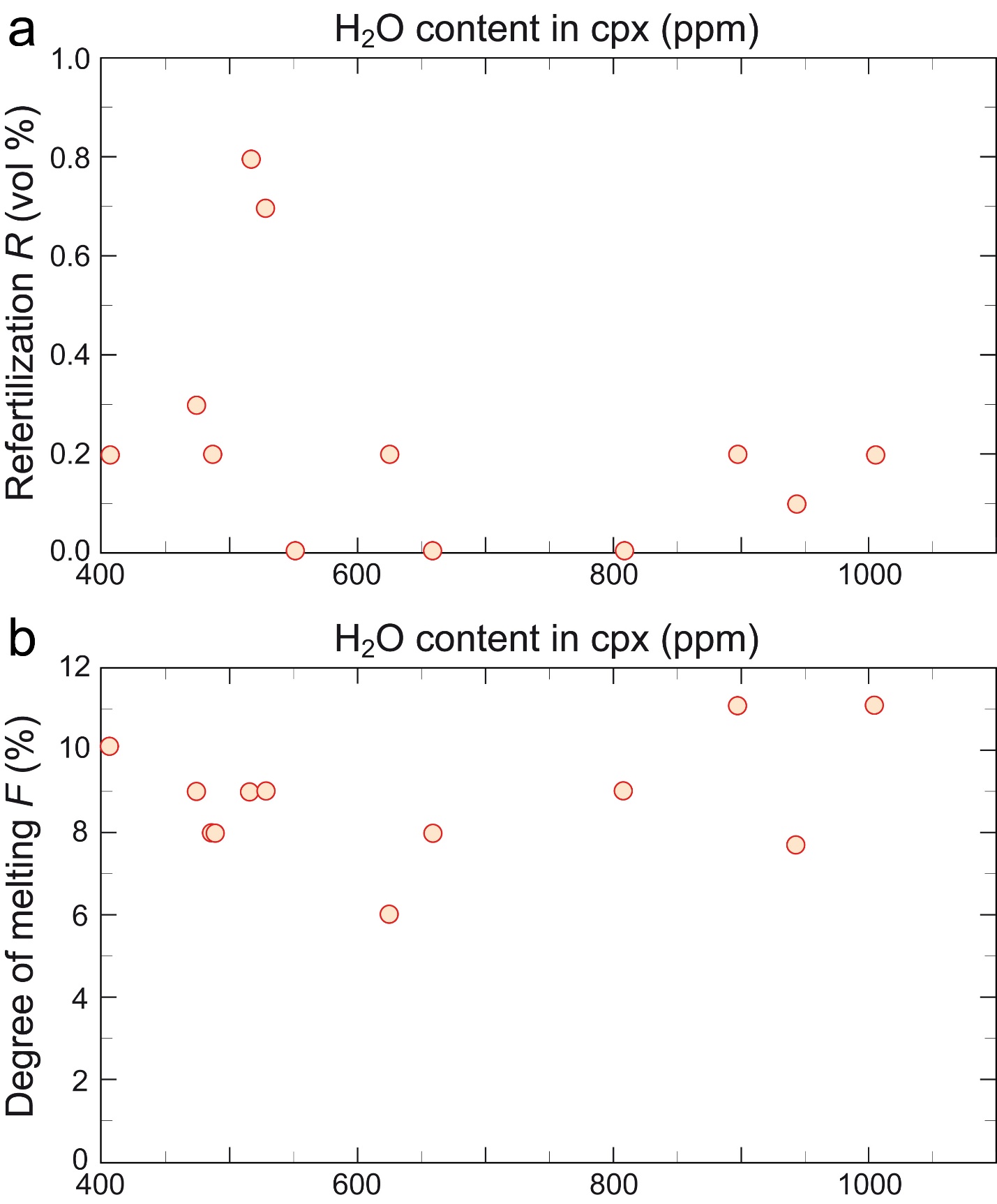
Supplementary Figure 6.** Influence of extent of melting and/or metasomatism on the H_2_O content of VLS residual peridotites. **a,** *R* is the amount of refertilization (vol. %). **b**, *F* represents the estimated mean degree of melting experienced by the VLS abyssal peridotites. Data from ref. 9.


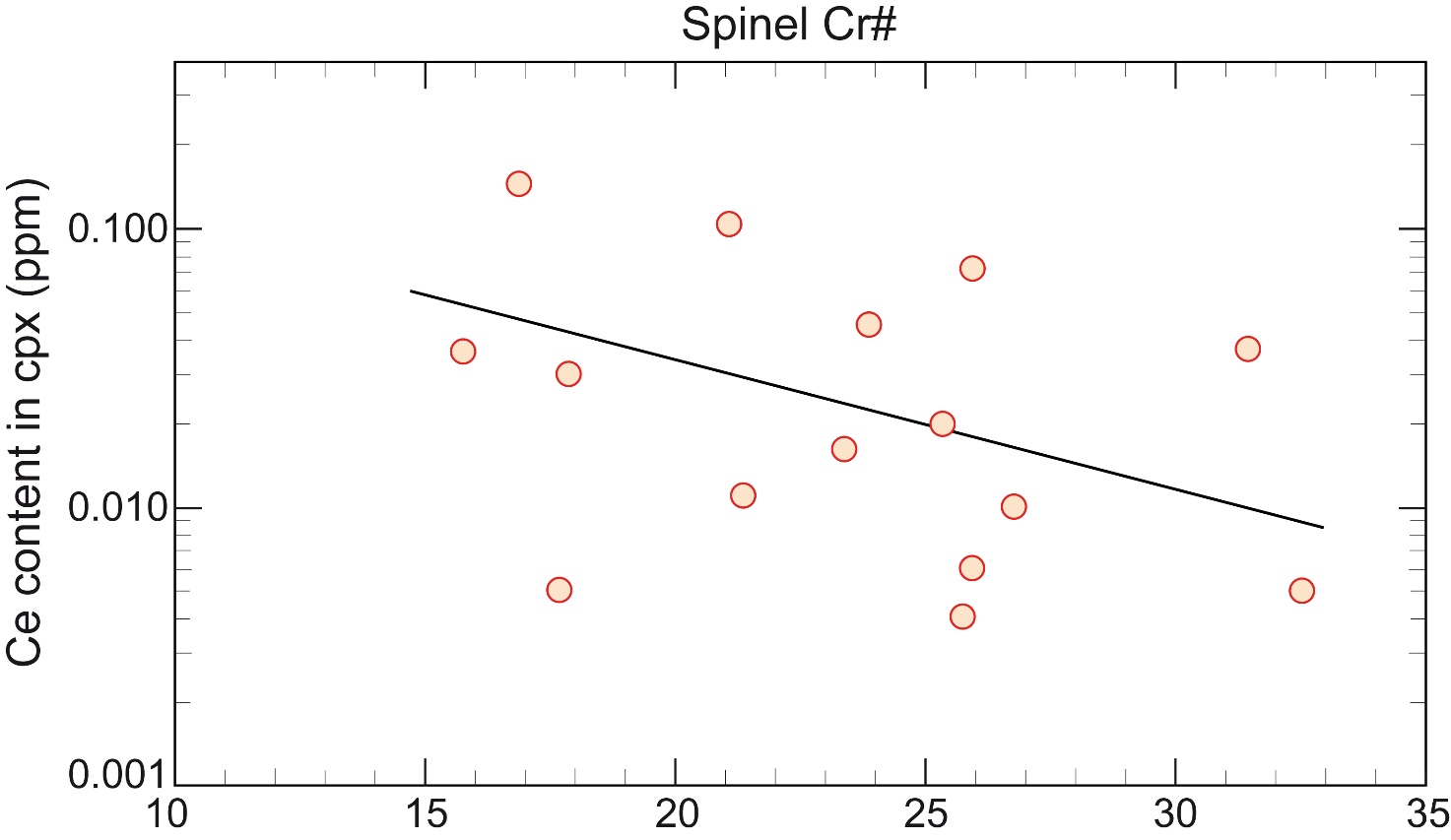


**Supplementary Figure 7.** Co-variations between Ce concentrations in cpx and Spinel Cr# (a proxy of mantle extent of melting). Note the expected inverse correlation between Ce and the degree of melting parameter. Regression line: black solid line. Data from ref. 9.

**Supplementary Table 1.** Vema abyssal peridotite δ^18^O contents. Laser fluorination oxygen isotope compositions of cpx and opx from the VLS peridotites.

| **Sample** | **δ^18^O cpx** | **δ^18^° opx** | **Δ^18^ opx-cpx** | |
| --- | --- | --- | --- | --- |
|  |  |  |  |  |
| **S1928-23** | 4,86 | 5,58 | 0,72 |  |
| **S2221-02** | 5,64 | 5,98 | 0,34 |  |
| **S2221-04** | 5,93 | 6,05 | 0,12 |  |
| **S2221-05** | 5,57 | 5,90 | 0,33 |  |
| **S1927-02** | 5,66 | 5,80 | 0,14 |  |
| **S1925-71** | 5,65 | 5,96 | 0,31 |  |
| **S1925-75** | 5,11 | 6,17 | 1,06 |  |
| **S1924-19** | 5,52 | 5,88 | 0,35 |  |
| **S1923-46** | 5,56 | 5,85 | 0,29 |  |
| **VE1-1** | 5,81 | 5,98 | 0,17 |  |
| **EW9305-15-23** | 5,52 | 5,80 | 0,28 |  |
| **EW9305-16-1** | 5,51 | 5,81 | 0,29 |  |
| **EW9305-17-5** | 5,64 | 5,83 | 0,19 |  |
| **S1904-76** | 5,57 | 5,88 | 0,31 |  |
| **S1904-77** | 5,38 | 5,98 | 0,59 |  |
| **S1913-03** | 5,82 | 6,11 | 0,28 |  |
| **S1913-36** | 5,62 | 5,96 | 0,34 |  |
| **S1904-33** | 5,24 | 5,52 | 0,28 |  |
| **S1904-42** | 5,21 | 5,37 | 0,16 |  |
| **S1905-111** | 5,36 |  |  |  |
| **S1912-08** | 5,82 | 5,93 | 0,11 |  |
| **S1915-71** | 4,88 |  |  |  |
|  |  |  |  |  |

Note: δ^18^O values result from averages of 2 measurements in samples with weight of 1-2 mg.

**Supplementary Table 2.** H_2_O contents in cpx and opx of abyssal peridotites and extent of melting parameters from published data^21,22,27,65^. Predicted H_2_O contents for melt in equilibrium with residual peridotites are estimated from opx and cpx water contents adopting partition coefficients $D_{H_{2}O}^{opx-melt}=0.019$ (value in bold) and $D_{H_{2}O}^{cpx-melt}=0.023$ (value in brackets) from refs.^38,39^.

| **Sample** | **H_2_O (ppm)** | | | | | **Spinel** | | **^$^F%** | **Melt H_2_O** | |  |
| --- | --- | --- | --- | --- | --- | --- | --- | --- | --- | --- | --- |
|  | **Opx** | | | | **Cpx** | **Cr#** | |  | **(wt%)** | |  |
| ***Hess Deep, East Pacific Rise (ref. 27)*** | | | | | | | | | | | |
| 147-895D-2R1-34-37 | | | | 124 |  | 53.0 | 18 | | **0.7** |  | |
| 147-895D-2R2-72-82 | | | | 233 |  | 52.0 | 17 | | **1.2** |  | |
| 147-895D-2R2-75-78 | | | | 230 |  | 51.0 | 17 | | **1.2** |  | |
| 147-895D-3R1-83-93 | | | | 229 |  | 52.0 | 17 | | **1.2** |  | |
| 147-895D-4R2-18-31 | | | | 86 |  | 54.0 | 18 | | **0.5** |  | |
| 147-895D-4R3-132-134 | | | | 102 |  | 52.0 | 17 | | **0.5** |  | |
| 147-895D-4R4-85-90 | | | | 88 |  | 53.0 | 18 | | **0.5** |  | |
| 147-895D-8R1-35-44 | | | | 164 |  | 54.0 | 18 | | **0.9** |  | |
| 147-895F-1R1-6-16 | | | | 108 |  | 53.0 | 18 | | **0.6** |  | |
| ***Southwest Indian Ridge, from 53°E to 63.5°E (ref. 65)*** | | | | | | | | | |  | |
| 21V-S9-D5-2 | | | | 59 |  |  |  | | **0.3** |  | |
| 21V-D1-1 | | | 24 | |  |  |  | | **0.1** |  | |
| 21V-D1-2 | | | 246 | |  | 26.2 | 11 | | **1.3** |  | |
| 21V-D1-3 | | | 93 | |  |  |  | | **0.5** |  | |
| 21V-S16-TVG4 | | | 171 | |  | 32.2 | 13 | | **0.9** |  | |
| Wb-18-b | | | 207 | |  | 16.5 | 6 | | **1.1** |  | |
| 20VII-S24-TVG20 | | | 257 | |  | 14.1 | 4 | | **1.4** |  | |
| 19III-S3-TVG2-4 | | | 262 | |  | 15.2 | 5 | | **1.4** |  | |
| ***Southwest Indian Ridge (ref. 22)*** | | | | | | | | | | | |
| PS86-6-38 | | | 171 | | 459 | 11.3 | 2 | | **0.9** (2.0) |  | |
| Van7-85-30 | | | 289 | | 793 |  |  | | **1.5** (3.4) |  | |
| Van7-85-42 | | | 145 | | 429 | 15.9 | 6 | | **0.8** (1.9) |  | |
| Van7-85-47 | | | 157 | | 415 | 17.4 | 7 | | **0.8** (1.8) |  | |
| Van7-85-49 | | | 198 | | 398 | 15.0 |  | | **1.0** (1.7) |  | |
| Van7-86-27 | | |  | | 748 |  |  | | (3.3) |  | |
| Van7-96-25 | | | 21 | | 458 | 13.8 | 4 | | **0.1** (2.0) |  | |
| Van7-96-28 | | | 122 | | 613 | 32.3 | 13 | | **0.6** (2.7) |  | |
| Van7-96-35 | | | 87 | | 413 | 24.0 | 10 | | **0.5** (1.8) |  | |
| Van7-96-38 | | | 116 | | 665 | 11.5 | 2 | | **0.6** (2.9) |  | |
| ***Gakkel Ridge, Arctic Ocean (ref. 22)*** | | | | | | | | | | | |
| PS59-235-17 | | 145 | | | 362 | 13.9 | 4 | | **0.8** (1.6) |  | |
| ***Mid Atlantic Ridge, ODP Leg 153 (ref. 21)*** | | | | | | | | | | | |
| 920D-15R1-8-15 | | | | 160 |  | 29.0 | 12 | | **0.8** |  | |
| 920D-22R4-104-109 | | | | 270 |  | 29.0 | 12 | | **1.4** |  | |

Note: Opx, orthopyroxenes; Cpx, clinopyroxenes and Sp, spinels.

^$^Extent of melting *F* estimated using sp Cr# according to ref. 19.

**^───────────────────────────────────────────────────────────────^**

**References**

1. Anders, E. & Grevesse, N. Abundances of the elements: Meteoritic and solar, *Geochim. Cosmochim. Acta* **53**, 197-214 (1989).
